# Improving Methods for Reporting Spatial Epidemiologic Data 

**DOI:** 10.3201/eid1408.080145

**Published:** 2008-08

**Authors:** A. Townsend Peterson

**Affiliations:** * University of Kansas, Lawrence, Kansas, USA

**Keywords:** County, ZIP code, census tract, spatial data, letter

## To the Editor

A recent perspective in this journal (*1*) pointed out problems with the present, county-referenced system for reporting spatial epidemiologic data. Problems identified included coarse spatial resolution of county-referenced data and differences across the United States in size of counties, making data for the western part of the country coarser in resolution than data for the eastern part. Eisen and Eisen correctly pointed out that these problems complicate spatial analyses of epidemiologic data (*1*). However, the solutions that they propose, referencing epidemiologic data to ZIP codes or census tracts, partially solve only the first problem.

The problem of regional differences in spatial resolution of county-referenced data is, unfortunately, reflected in counties, ZIP codes, and census tracts, as shown in plots of nearest-neighbor distances among unit centroids as a function of longitude (Figure). Because all 3 regionalizations are based on human populations, the much greater population density in the eastern United States creates finer scale dispersion in the east. Thus, a shift to ZIP codes or census tracts does nothing to resolve the problem of regional differences in spatial resolution.

**Figure:**
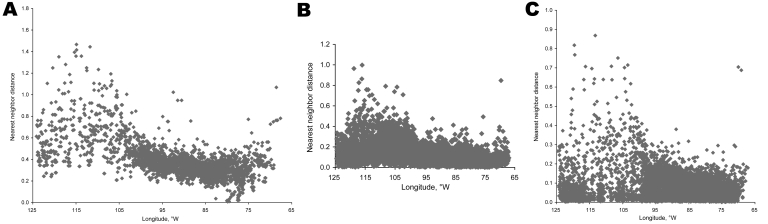
Longitudinal patterns in nearest-neighbor distances for A) counties, B) ZIP codes, and C) census tracts across the lower 48 United States, showing trends toward greater spacing among districts in the western United States compared with the eastern United States in all 3 regionalizations.

The problem of coarse spatial resolution is only partially addressed by the ZIP code or census tract solution. ZIP codes and census tracts cover fixed areas and can misrepresent the spatial precision of epidemiologic records. A traveling salesperson who covers the state of Wyoming each week would be represented identically as his or her next-door neighbor who is housebound, although spatial precision differs considerably between the 2 persons. Precision of the housebound neighbor could be better represented than county, ZIP code, or census tract. ZIP codes and census tracts change periodically, and ZIP codes do not have defined spatial extents per se (*2*). Thus, a better and more flexible solution is needed.

The biodiversity world has already addressed this challenge. The point-radius method for georeferencing locality descriptions (*3*) estimates a best guess for the exposure site (e.g., residence, workplace) but describes uncertainty in that georeference is a radius that expresses spatial uncertainty in the record (i.e., compare our traveling salesperson with his or her housebound neighbor) and in translation into geographic coordinates (including uncertainty in the locality descriptor, spatial footprint of the locality described, imprecision in the locality identified, and any other sources of imprecision). Point-radius georeferences are easily recorded and reported, are consistent and reproducible, and are more precise and considerably more stable than ZIP codes or census tracts.

As an example of how the point-radius method would be applied, the locality for our traveling salesperson would be assigned to his or her house, but the error radius would be 360 km (based on the corner-to-corner distance across Wyoming). The housebound neighbor might have a similar set of coordinates (next door), but the error radius might be 0.1 km (breadth of the house plus the imprecision of the global positioning system unit). When a researcher uses these data, he or she might wish to analyze occurrence of this disease with a spatial precision of 1 km; e.g., applying a filter to exclude those data records too imprecise for this study, he or she would exclude the data record for the salesperson (because the salesperson may have contracted the disease in another sector of the state) but include that for the housebound neighbor. Alternatively, the researcher may include variable degrees of precision in the analysis according each to record a precision or certainty corresponding to its error radius, as in recent spatial analyses of Marburg virus transmission risk (*4*) and climate change effects on plague and tularemia transmission (*5*).

How specifically would this method be implemented in public health surveillance? If data are to be captured initially on paper, the data recorder would simply record the focal point of the person’s activities (usually a residence) and an approximate description of the person’s movements (e.g., broadly across the state, housebound, within 20 miles). These descriptions are easily georeferenced post hoc by using recently developed software tools (e.g., Biogeomancer, www.biogeomancer.org/). A more promising solution, if initial data capture is electronic, would be adaptation of some of these software solutions to the public health challenge. A flexible-resolution map with political boundaries, named places, and roads and streets could enable immediate digitization of the central point and the error radius even during direct consultation with the patient (when feasible).

The point-radius approach is novel to most epidemiologic applications but offers considerable advantages. When fine-resolution data are available, researchers will have this more precise information and can distinguish it from coarser resolution data; when actual data are coarser, this information is also expressed. Researchers will be able to filter epidemiologic occurrence information to retain those data that are sufficiently precise for particular applications, thus offering a considerable improvement over any of the 3 polygon-based approaches (ZIP codes, census tracts, and counties). Thus, the recent publication cited (*1*) got the question right but the answer wrong.
