# Supplementary material for: Improving Methods for Reporting Spatial Epidemiologic Data
Source: Emerg Infect Dis. 2008 Aug;14(8):1335–7. doi: 10.3201/eid1408.080145 (PMC2600409; doi:10.3201/eid1408.080145)
Supplement: Related texts

# Reply: In Response

**Authors:** Lars Eisen, Rebecca J. Eisen

**Affiliations:** * Colorado State University, Fort Collins, Colorado, USA; † Centers for Disease Control and Prevention, Fort Collins

In his comment, Peterson reiterates the need for improved methods for collecting and presenting spatial epidemiologic data for vector-borne diseases (*1*). He agrees with us that lack of reliable data on probable pathogen exposure site is an obstacle to the development of predictive spatial risk models (*2*). In that article we noted, “New methods are urgently needed to determine probable pathogen exposure sites that will yield reliable results while taking into account economic and time constraints of the public health system and attending physicians.” Peterson suggests that the point-radius method is a viable solution to this problem. Unfortunately, its practical implementation for vector-borne diseases is neither reliable nor cost-effective.

With regard to practical implementation of the point-radius method in a public health setting, Peterson states, “If data are to be captured initially on paper, the data recorder would simply record the focal point of the person’s activities (usually a residence) and an approximate description of the person’s movements (e.g., broadly across the state, housebound, within 20 miles)” (*1*). We find a number of serious problems with this approach to determining probable sites of pathogen exposure, primarily that meaningful use of the point-radius method 1) will require not only recording detailed movements during the perceived window of opportunity for pathogen exposure but also weighting of risk by activity type and, for some vector-borne diseases, time of day; and 2) will require the public health community to allocate resources to in-depth interviews conducted by specially trained personnel.

Our first concern is that Peterson’s scenario does not distinguish between a car trip to the mall at noon and spending an evening on the golf course. In reality, one activity presents minimal risk for exposure to mosquitoes infected with West Nile virus, whereas the other is a potential high-risk activity. Giving equal weight to the movements represented by these activities will assuredly produce an unreliable result for probable pathogen exposure site. Other issues are patient recall and reluctance to provide information on movement patterns and specific activities. Peterson’s suggestion that the data recorder would simply record the focal point of the person’s activities and an approximate description of the person’s movements is therefore a grossly oversimplified solution to a complex public health problem.

With regard to the second concern, the average physician likely lacks the knowledge, time, and training in vector-borne disease epidemiology and ecology needed to accurately assess when and where risk for pathogen exposure occurred. To be of use, the method will require in-depth patient interviews by specially trained personnel from local or state health departments. Even then, we doubt that the quality of data gleaned would justify the cost incurred.

We fail to see that the quality of information gathered by using the point-radius method would be an improvement over our suggestion. In our original article, we suggested using sets of standardized questions that are tailored to a given vector-borne disease. We also indicated that a critical minimal need includes a basic assessment of whether pathogen exposure likely occurred in 1) the peridomestic environment, 2) outside the peridomestic environment but within the county of residence, or 3) outside the county of residence (*2*).

The challenge of how to most effectively collect and present spatial epidemiologic data is neither conceptual nor technologic; rather, it is logistic and legal. Any new method must 1) weigh the public health utility of the method against the time and cost required for the public health system to implement it and 2) comply with existing patient privacy laws. The point-radius method clearly fails on the first count and also likely will present substantial problems in terms of patient privacy.

We agree that presenting data for case counts and disease incidence by ZIP code or census tract falls short of the desired level of spatial precision. However, this realistic compromise 1) is a marked improvement over the current practice to display only county-based spatial patterns for case counts or incidence; 2) incurs only minimal added time and cost for the public health community; and 3) can be implemented, especially for census tracts, with minimal concerns regarding patient privacy.

## References

[1] Peterson AT. Improving methods for reporting spatial epidemiologic data [letter]. Emerg Infect Dis. 2008;14:1335–6. 10.3201/eid1410.08016918680681PMC2600409

[2] Eisen L, Eisen RJ. Need for improved methods to collect and present spatial epidemiologic data for vectorborne diseases. Emerg Infect Dis. 2007;13:1816–20.1825802910.3201/eid1312.070211PMC2876744
